# Dual-Energy Computed Tomography Collagen Density Mapping of the Cranio-Cervical Ligaments—A Retrospective Feasibility Study

**DOI:** 10.3390/diagnostics12122966

**Published:** 2022-11-27

**Authors:** Thomas Matthias Wittig, Katharina Ziegeler, Virginie Kreutzinger, Milen Golchev, Simon Ponsel, Torsten Diekhoff, Sevtap Tugce Ulas

**Affiliations:** 1 Department of Radiology, Charité-Universitätsmedizin Berlin, Campus Mitte, Humboldt-Universität zu Berlin, Freie Universität Berlin, 10117 Berlin, Germany; 2 Department of Radiology, Vivantes Klinikum im Friedrichshain, 10249 Berlin, Germany; 3 Berlin Institute of Health at Charité-Universitätsmedizin Berlin, 10117 Berlin, Germany

**Keywords:** dual-energy CT, ligaments, atlanto-axial joint, collagen, chondrocalcinosis

## Abstract

The objectives of this study were to investigate the mean collagen content of the atlanto-axial joint (AAJ) ligaments in a cohort without inflammatory disease and to analyze clinical confounders such as age, sex, and presence of ligamentous calcifications. A total of 153 patients who underwent dual-energy computed tomography (DECT) due to various reasons (e.g., suspected cancer or infection) were included in this retrospective study. Reconstruction of collagen density maps from the DECT dataset was performed. Region of interest (ROI) analysis was performed to assess densities in the following regions: ligamentum transversum atlantis (LTA), ligamenta alaria, fasciculi longitudinales, ligamentum nuchae, and retro-odontoid soft tissue (RDS). Osteoarthritis (OA) and the presence of calcifications were assessed by two experienced readers blinded to clinical data. Subgroup comparisons were performed using unpaired t-tests. The correlation of collagen density and clinical factors was investigated using Pearson’s correlation coefficient. Mean LTA collagen density was 141.7 (SD 35.7). Ligamentous calcifications were rare (14.4 %). OA of the AAJ was common (91.5 %). LTA collagen density was not associated with age (Pearson’s r of 0.109; *p* = 0.180) and was not significantly higher in patients with OA (*p* = 0.070). No correlations between RDS thickness, collagen density or calcifications were found. Our results show collagen density mapping of the cranio-cervical joint ligaments to be feasible; collagen densities are not significantly associated with age, sex, AAJ degeneration, or asymptomatic ligamentous calcification.

## 1. Introduction

Due to its anatomical position and structure, the atlanto-axial joint (AAJ) has special significance as a link between the bony skull and the spinal column. The joints of the head allow finely tuned movement mechanics around three axes with extension, flexion, lateral flexion, and rotation of the head. Degenerative changes are frequently found at this particularly stressed joint, which usually become apparent as an incidental finding during radiological examinations [1]. Calcifications of the atlanto-axial ligaments, especially the ligamentum transversum atlantis (LTA), are a very common finding in elderly patients. A previous retrospective study showed an incidence of up to 49% in patients over 80 years of age in a cohort of patients with CT imaging obtained for acute trauma of the cervical spine [2]. Such calcifications of the AAJ are associated with calcium pyrophosphate dihydrate deposition disease (CPPD) [2] and are often found as an incidental imaging finding in asymptomatic patients during radiological imaging without further clinical consequences, diagnostic testing, or need for subsequent therapy applications [3]. However, in rare cases, they can also cause acute inflammation, which is defined as crowned dens syndrome, with symptomatic presentation including neck pain, fever, and the elevation of inflammatory blood markers [4]. In general, several studies showed patients with primary hyperparathyroidism to have an up to four times higher risk of CPPD, most likely caused by an increased amount of calcium and/or parathyroid hormone due to metabolic bone diseases [5,6,7,8,9]. Furthermore, risk of CPPD seems to be associated with hypomagnesaemia and possibly associated with haemochromatosis [3], but more evidence is needed in this aspect.

Apart from their possible role in inflammatory disease, ligamentous calcifications in this anatomical area may carry special biomechanical significance as they are associated with a higher rate of unstable dens fractures (Anderson and D’Alonzo type II) after minor trauma [10]. However, the specific remodeling processes of the extracellular matrix of the ligaments are not fully understood.

A promising tool for the non-invasive study of such processes is dual-energy computed tomography (DECT), which has seen a multitude of technical advances [11] over the last few years. Material-specific attenuations in low- and high-energy spectra [12] allow for a relatively specific characterization of the material composition within a certain object [13]. In musculoskeletal imaging, this technique is mainly used to detect uric acid crystal depositions in gout arthropathy [14]. In addition, DECT allows a differentiated quantification of bone marrow edema using virtual non-calcium images [15]. Furthermore, it also allows one to generate specific collagen density maps [16,17]. This enables a non-invasive quantification of collagen density based on specific radiation attenuation at different energy levels. In a recent study of our group, we showed a significantly increased collagen density in the wrist ligaments in patients with CPPD versus a control cohort using this technique [17].

Changes in collagen density may indicate altered biomechanical properties of the affected ligaments. Thus, not only the knowledge of inflammatory diseases of the AAJ but also the role of the LTA in dens fractures after minor trauma can potentially be further elucidated using this technique. The aim of this work is therefore to investigate the mean collagen content of the ligaments of the AAJ in a cohort without inflammatory disease, in order to establish a baseline and to investigate clinical confounders such as age, sex, and presence of ligamentous calcifications.

## 2. Materials and Methods

Approval from the institutional ethics review board was obtained prior to study commencement (EA1/247/21). All patients gave their written informed consent for the scientific use of their imaging and clinical data prior to the DECT scan, which is standard protocol in our institution. All procedures performed in studies involving human participants were in accordance with the ethical standards of the institutional and/or national research committee and with the 1964 Helsinki Declaration and its later amendments, or comparable ethical standards. 

### 2.1. Subjects

All patients who underwent a DECT scan of the neck with clinical indication between May 2019 and August 2020 in the Department of Radiology of our university hospital were included in this retrospective analysis. Patients with known rheumatic joint diseases or hyperparathyroidism were excluded, as well as patients with insufficient image quality for the application of further reconstruction algorithms. Since most patients received their DECT of the neck due to their oncologic history, information regarding previous radiation and chemotherapy was also collected.

### 2.2. Imaging Technique

All patients underwent a DECT scan of the neck in a 320-row single-source computed tomography (CT) scanner (Canon Aquilion One Vision, Canon Medical Systems) with sequential volume acquisition of two different energy datasets (135 and 80 kVp with 180 mA and 500 mA). A split-bolus contrast medium injection protocol was used (body weight-adapted Ultravist 370 (Bayer), 1 mL/kg). The scan of the neck was performed with a delay of 8 s after the start of the second bolus injection in volume mode with a z-axis coverage of 16 cm without table movement. Rotation time was 0.5 s. Collagen density maps were calculated using a vendor software (Canon Medical Systems) on the CT console, applying a dual-energy gradient of 1.1 for collagen on the three-material decomposition software, and were reconstructed with 0.5 mm slice thickness. All images were pseudonymized. 

### 2.3. Region of Interest Analysis

**Figure 1:**
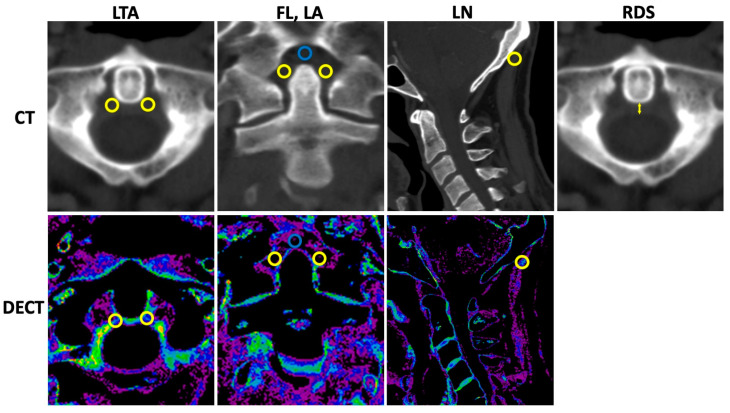
Measurement of collagen density and retro-odontoid soft tissue. CT = computed tomography, DECT = dual-energy CT, LTA = ligamentum transversum atlantis, FL = fasciculi longitudinalis, LA = ligamenta alaria, LN = ligamentum nuchae, and RDS = retro-odontoid soft tissue thickness. Upper row indicates CT with 135 kVp reconstructions. Lower row refers to DECT-based collagen maps. Region of interest (ROI) measurements were performed with predefined size of 9.5 mm^2^ for all measurements using the collagen density maps. In total, twelve ROI measurements were performed (six in 135 kVp images, six in collagen maps) as follows: LTA (two ROIs per reconstruction in axial orientation (left and right orientation, yellow ROIs); FL (one ROI per reconstruction in coronal orientation, blue ROI); LA (two ROIs per reconstruction in coronal orientation, yellow ROIs); and LN (one ROI per reconstruction in sagittal orientation, yellow ROI). Thickness of the retro-odontoid soft tissue (indicated by the yellow arrow) was measured using the 135 kVp CT reconstruction in axial orientation.

Standardized region of interest (ROI) analysis was performed by a specially trained research student (TMW) using the collagen density maps reconstructed from the DECT datasets and separately for the LTA; both ROIs were used for calculation of mean, the fasciculi longitudinales, and for the ligamenta alaria with dedicated software (Horos v.2.2.0, The Horos Project). The collagen density of the ligamentum nuchae served as reference. A size of 9.5 mm^2^ was chosen for all ROI measurements. The mean densities (Hounsfield unit, HU) and standard deviations (SDs) were collected. Furthermore, only the thickness of the retro-odontoid soft tissue was measured using the 135 kVp CT reconstruction in axial orientation. Imaging examples with illustrations of standardized ROI placement and imaging examples of the retro-odontoid soft tissue measurement are provided in Figure 1.

### 2.4. Image Reading

**Figure 2:**
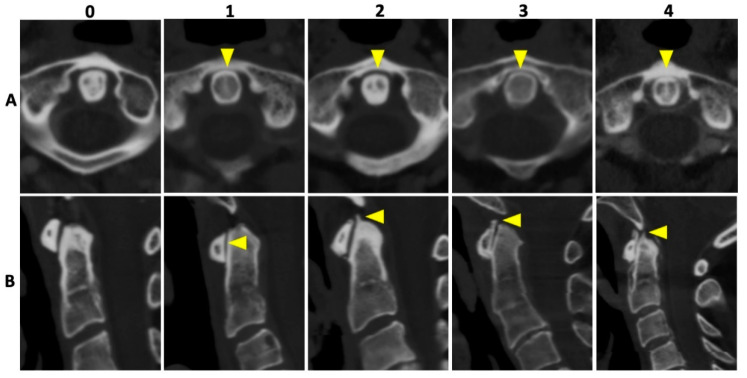
Imaging examples of osteoarthritis of the atlanto-axial joint. Imaging examples of five different patients according to the Kellgren and Lawrence [18] grading of osteoarthrosis (OA). Upper row (**A**) with axial orientation of 135 kVp images, and lower row (**B**) with sagittal orientation of 135 kVp images, respectively. From left to right (both rows) an increase in OA grading with no OA (0), beginning OA (1), minimal OA (2), moderate OA (3), and severe OA (4) of the atlanto-axial joint is shown (yellow arrowheads).

**Figure 3:**
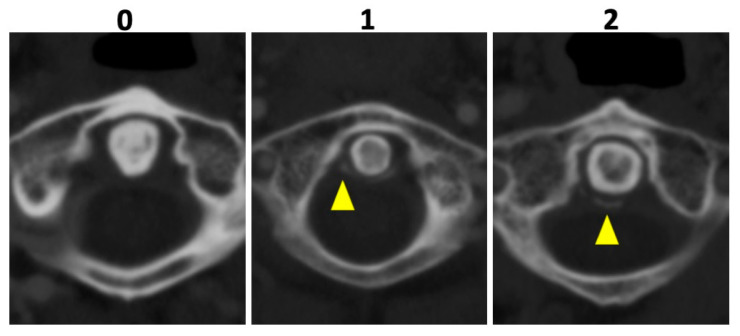
Imaging examples of ligament calcification grading. Imaging examples of three different patients according to the assessment of ligament calcifications of the ligamentum transversum atlantis based on axial-orientated 135 kVp image reconstructions. Grading was performed as follows: absence of calcifications (0), punctuate calcifications (1), and confluent calcifications (2) (yellow arrowheads).

In all patients, 135 kVp CT reconstructions were assessed separately by two readers who were blinded to all clinical data: VK with 5 years of experience and MG with 7 years of experience in musculoskeletal imaging (MSK). Both readers assessed osteoarthritis (OA) of the AAJ categorically, using the Kellgren and Lawrence score (KL); for imaging examples per category see Figure 2 [18]. Furthermore, calcifications of the LTA were graded as absent (=0), punctuate (=1), or confluent (=2) and, if present, as limited, moderate, or extensive. Imaging examples are presented in Figure 3. Cases of disagreement were resolved by an experienced MSK radiologist (KZ). On the patient level, individuals with an OA grading of ≥2 were considered positive for OA for the respective region. 

### 2.5. Statistical Analysis

Statistical analysis was performed using SPSS (Version 28). Subgroup comparisons were performed with unpaired t-tests and associations between collagen density and clinical factors were investigated using Pearson’s correlation coefficient. Agreement between readers was assessed using intra-class correlation coefficients (ICC) with a two-way mixed model.

## 3. Results

### 3.1. Subjects

**Figure 4:**
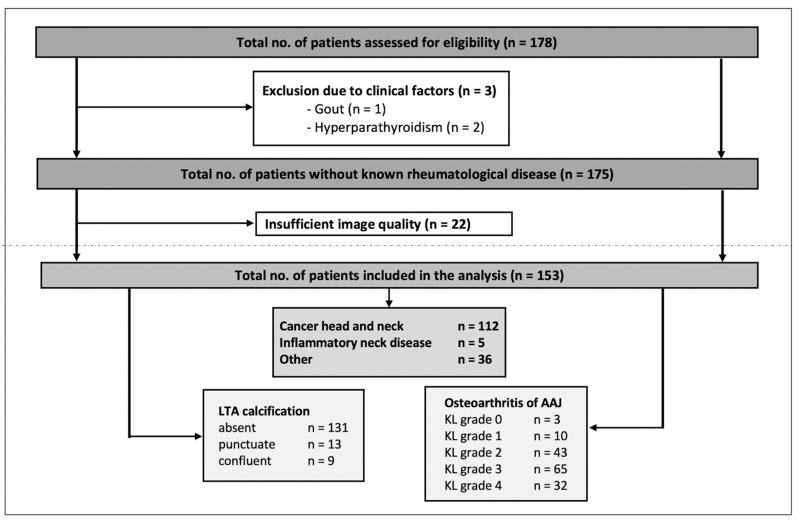
Flowchart of the study inclusion and results of the scoring. LTA = ligamentum transversum atlantis, AAJ = atlantoaxial joint, KL = Kellgren and Lawrence grading.

The number of patients evaluated for inclusion was 178—of these, 3 patients were excluded because of clinical factors (gout or hyperparathyroidism) and 22 patients were excluded because of insufficient image quality. In the case of multiple DECT exams (applicable in 16 cases of 15 patients), only the first exam was included for analysis. Thus, 153 patients (124 men, 29 women) with a mean age of 65 years (SD 12, range 28–88 years) were included in the final analysis. Imaging indication and respective known diseases of the patients were the following: head and neck cancer (e.g., laryngeal cancer, oropharyngeal cancer, and hypopharyngeal cancer), inflammatory neck disease (e.g., acute tonsillitis, cervical phlegmon, and postoperative imaging after tonsillectomy), and others (e.g., other tumor diseases such as thyroid carcinoma, esophageal carcinoma, malignant melanoma, or cervical lymphadenopathy of unknown cause), respectively. The study population and the results of the scoring are presented in Figure 4, Table 1 and Supplementary Table S1.

### 3.2. Descriptive Results

Mean collagen density of the LTA was 141.7 (SD 35.7; range 22.8–223.0). Mean collagen density of the fasciculi longitudinales (FL), ligamenta alaria (LA), and ligamentum nuchae (LN) were as follows: 62.8 (SD 39.9; range 13.5–209,9), 117.3 (SD 37.2; range 4.3–214,9), 110.6 (SD 43.0; range 20.2–237.0). The distribution of the measured collagen densities at the different locations is presented in Supplementary Figure S1. The mean thickness of the retro-odontoid soft tissue was 3.5 mm (SD 0.8; range 1.91–6.34 mm). 

Ligamentous calcifications were rare and only detected in 22/153 (14.4%) patients; of these, 13 exhibited punctuate and 9 exhibited confluent calcifications. OA of the AAJ was a common finding: 13/153 (8.5%) received a KL grade of <2 and 20.9% (32/153) received a KL grade of 4. In total, OA (KL ≥ 2) was found in 91.5 % (140/153) (see also Figure 4). 

### 3.3. Factors of Influence on Collagen Density

In our cohort, age was not significantly associated with collagen density of the LTA, shown by the weak Pearson’s r of 0.109 (*p* = 0.180). Furthermore, sex also showed no correlation with collagen density of the LTA (Pearson´s r of 0.264; *p* = 0.167). In patients with osteoarthritis (KL ≥ 2), the collagen density was not significantly higher compared to patients without relevant degenerative changes (KL < 2), with a mean collagen density of 140.3 (SD 36.0) versus 156.9 (SD 28.8), respectively, shown by the Levene test (*p* = 0.070). Collagen density did not differ significantly in patients with or without calcifications (149.6, SD 35.7 versus 140.4, SD 35.6; *p* = 0.269), but there was overall slightly higher observed collagen density in patients with calcifications. Only a slight trend of correlation was found between the collagen density and retro-odontoid soft tissue thickness, shown by the weak Pearson´s r of 0.170 (*p* = 0.036). The retro-odontoid soft tissue thickness was not observed to increase significantly in patients with calcifications, with a mean of 3.7 mm (SD 0.8 mm) versus 3.4 mm (SD 0.8 mm) in patients without calcifications (*p* = 0.258).

### 3.4. Image Reading

Agreement between readers for OA of the AAJ was moderate with an ICC of 0.54 (95% confidence interval (CI) 0.37–0.66; *p* < 0.001), while agreement regarding calcifications was good with an ICC of 0.83 (95% CI 0.37–0.95; *p* < 0.001).

## 4. Discussion

Our analysis marks the first attempt at a description of collagen densities of the ligaments of the cranio-cervical junction (CCJ) as detected by dual-energy computed tomography. The measured collagen densities showed a wide range but were not significantly associated with age, sex, or osteoarthritis of the CCJ.

The ligaments of the CCJ, such as the LTA or the LA, are responsible for the complex transformation and transmission of forces from the skull to the spine. In addition to the transmission from skull to spine, a major biomechanical focus of the LTA and LA and other cranio-cervical ligaments is to provide a full range of motion and significant mobility [19]. We assumed that collagen density is increased in patients with OA. Based on our results showing no significant association of collagen densities and degeneration, this hypothesis could not be confirmed. 

Collagen density maps allow the non-invasive quantification of microstructural changes. To the best of our knowledge, only few studies have investigated this method. In a recent study, this technique was successfully assessed to evaluate post-operative changes in tendon grafts after knee ligament reconstruction [20]. In a further study, collagen density maps of the wrist ligaments in a clinical CPPD cohort were evaluated and compared to a healthy control group [17]. This study showed a higher collagen density in the symptomatic CPPD cohort compared to the healthy control group. In contrast to our cohort without known inflammatory disease, this supports the hypothesis that changes in collagen density actually require an inflammatory process [21,22] and are less common in asymptomatic depositions [3]. 

CPPD, as the third most common inflammatory arthritis [23], is associated with radiologic features such as more frequent and severe osteophytes [22,24], and is also associated with more inflammatory features and more rapid progression [21] compared to OA without CPPD. Our results show fewer ligamentous calcifications than previous investigations [2]. Chang et al. report more frequent calcifications and report association with age, but also with increased thickening of the retro-odontoid soft tissue [2], which was not the case in our study. The absence of a large number of elderly patients and a small number of female patients may apply as presumed causes for the differences. This may explain why the presence of calcification in our study was not associated with an enlargement in the retro-odontoid soft tissue.

DECT is a promising imaging technique to evaluate changes in ligamentous collagen densities in patients suffering from crowned dens syndrome [25] or in patients with dens axis fracture, e.g., Anderson d´Alonzo type II [10]. Johnson et al. presented DECT for the first time for the reliable visualization of collagen [16], and following various studies have demonstrated DECT-based collagen analysis of ligaments of the hands [17,26], feet [26], and knees [27] to be a feasible application. Symptomatic patients with crowned dens syndrome and acute onset of cervical pain may benefit from DECT-based analysis by collagen mapping due to the increased risk of pseudogout of the cervical spine [28]. Thus, depending on the collagen measurement, therapeutic consequences may be drawn and management may be adapted towards individualized patient-centered management. DECT-based collagen analysis allows the quantitative assessment of follow-up and the re-evaluation of respective medical or interventional treatments in the course of the therapeutic process. A further clinical implication is patients with dens axis fractures of various causes. The use of quantitative collagen analysis in the evaluation of microstructural changes enables monitoring over time with regard to the presence of pseudoarthrosis and the possible prediction of secondary complications due to the underlying hypothesis of a higher risk of secondary complications in patients with pseudarthrosis of a dens axis fracture. 

Some limitations need to be discussed. The patient cohort shows a limited sample size and was recruited in a retrospective study design. It remains to be discussed whether the inclusion of a larger patient population would have resulted in significantly higher collagen density in OA. Furthermore, this cohort represents a group of patients without known inflammatory disease of the CCJ, heterogenous primary cause, and indication for imaging. In addition, only few patients (22/153) showed ligamentous calcifications, which may limit generalizability. In addition, changes in collagen density that may occur after radiation therapy have not been considered separately in this analysis. Furthermore, the reconstruction of collagen density maps was performed using postcontrast DECT datasets. This might also have influenced the measured collagen density. Due to the retrospective study design, a histological reference standard is missing. To verify and validate these observations, further research and prospective clinical studies are needed. 

In conclusion, our results show that collagen density mapping of the ligaments of the CCJ is feasible; the measured collagen densities were not significantly associated with age, sex, degeneration of the AAJ, or asymptomatic ligamentous calcification. The use of DECT-based collagen analysis in patients with CPPD and crowned dens syndrome or dens axis fractures is a promising tool, but further evaluation is needed.

## Figures and Tables

**Table 1:** Characteristics of the study population.

Characteristics	
Number of patients (women/men)	153 (29/124)
Mean age (y) (SD; range)	65 (12; 28–88)
Number of patients with radiation therapy	64
Number of patients wth chemotherapy	44

## Data Availability

The data presented in this study are available on request from the corresponding author.

## Supplementary Materials

The following supporting information can be downloaded at https://www.mdpi.com/article/10.3390/diagnostics12122966/s1, Figure S1: Quantitative assessment of the mean collagen density in the different locations. LTA = ligamentum transversum atlantis, FL = fasciculi longitudinalis, LA = ligamenta alaria, LN = ligamentum nuchae, HU = Hounsfield units; Table S1: Patient cohort characteristics.
