# Supplementary material for: Dual-Energy Computed Tomography Collagen Density Mapping of the Cranio-Cervical Ligaments—A Retrospective Feasibility Study
Source: Diagnostics (Basel). 2022 Nov 27;12(12):2966. doi: 10.3390/diagnostics12122966 (PMC9776840; doi:10.3390/diagnostics12122966)
Supplement: Supplementary file 1 [file diagnostics-12-02966-s001.zip › Supplementary Table S1.pdf]

Supplementary Table S1: Patient cohort characteristics.

IMAGING INDICATION	THERAPY	
	Radiatio<br>Total n = 64	Chemotherapy<br>Total n = 44
Cancer Head & Neck (n = 112)		
- Laryngeal cancer (n = 50)	n = 24	n = 14
- Oropharyngeal cancer (n = 23)	n = 14	n = 11
- Hypopharyngeal cancer (n = 9)	n = 6	n = 5
- Tonsillar carcinoma (n = 7)	n = 3	n = 1
- Tongue base carcinoma (n = 4)	n = 3	n = 3
- Other (n = 19)	n = 8	n = 4
Inflammatory Neck Disease (n = 5)		
- Post abscess tonsillectomy (n = 2)	n = 0	n = 0
- Cervical phlegmon (n = 2)	n = 0	n = 0
- Acute tonsillitis with peritonsillar abscess (n = 1)	n = 0	n = 0
Other (n = 36)		
- Malignant melanoma (n = 3)	n = 0	n = 0
- Lung cancer (n = 2)	n = 0	n = 1
- Other (n = 31)	n = 6	n = 5
