# Supplementary figures and images for: Dual-Energy Computed Tomography Collagen Density Mapping of the Cranio-Cervical Ligaments—A Retrospective Feasibility Study
Source: Diagnostics (Basel). 2022 Nov 27;12(12):2966. doi: 10.3390/diagnostics12122966 (PMC9776840; doi:10.3390/diagnostics12122966)

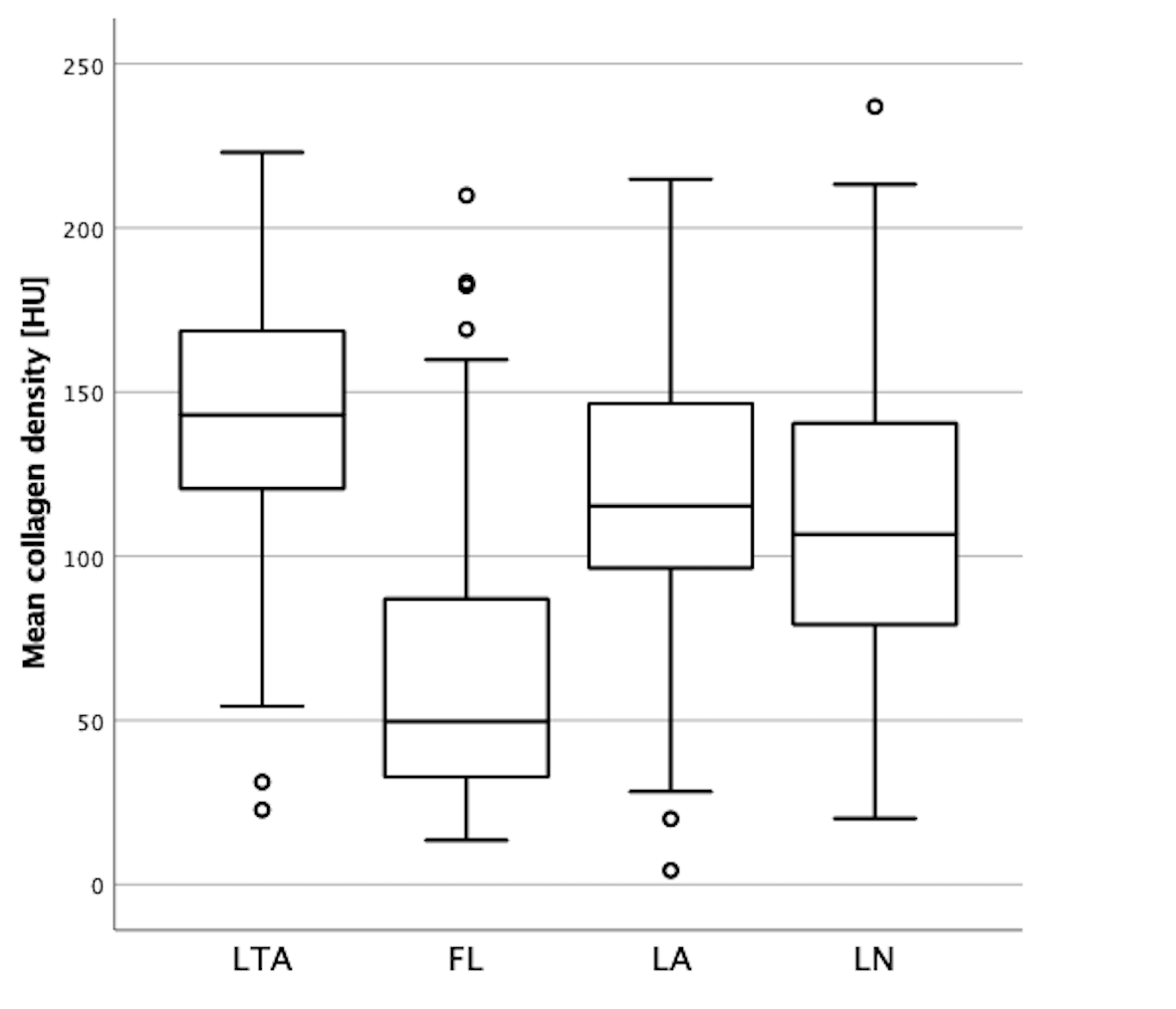

Supplement: Supplementary file 1 [file diagnostics-12-02966-s001.zip › Supplement Figure S1.tif]
